# Supplementary material for: Atomistic ensemble of active SHP2 phosphatase
Source: Commun Biol. 2023 Dec 21;6:1289. doi: 10.1038/s42003-023-05682-5 (PMC10739809; doi:10.1038/s42003-023-05682-5)
Supplement: Supplementary file 2 — Description of Additional Supplementary Files [file 42003_2023_5682_MOESM2_ESM.pdf]

Description of Additional Supplementary Files

File name: Movie S1
Description: Typical trajectory of the roto-translation of the C-SH2 domain relative to PTP in the truncated SHP2 DN-SH2, as described by principal components PC1 and PC2. Principal components were calculated from the rigid core of the C-SH2 domain after least-square fitting over the core of the PTP domain. The C-SH2 domain is colored in orange, the PTP domain in pink.

File name: Movie S2
Description: Exemplary structures derived from the solution ensemble of the constitutively active SHP2E76K mutant. The solution ensemble of SHP2E76K comprises a set of conformational arrangements where the N-SH2 domain is either close to the surface of the PTP domain or completely detached from PTP.

File name: Movie S3
Description: Small-angle X-ray scattering (SAXS) curve calculated from the MD ensembles of the first 500 most populated clusters of SHP2E76K (red line). For comparison, the experimental curve (black dots), reported as raw data, and the single-structure model by Pádua et al. (green line)2 are shown. Radii of gyration (Rg) from the MD ensembles and from experiment are reported with black and red font, respectively. The Rg values from MD ensembles were obtained by Guinier fits to calculated SAXS curves, thus the values include contributions from the hydration layer.
